# Sampling and processing blood samples within the South American Youth/Child cARdiovascular and Environmental (SAYCARE) Study

**DOI:** 10.1038/s41598-020-57457-1

**Published:** 2020-01-20

**Authors:** Graciela López, Raphael Assali Serruya, Magalí Barchuk, Diego Gaitan-Charry, Francisco Leonardo Torres-Leal, Luis Alberto Moreno, Carlos Alberto Delgado, Heráclito Barbosa Carvalho, Augusto César Ferreira De Moraes, Gabriela Berg

**Affiliations:** 1 Universidad de Buenos Aires, Facultad de Farmacia y Bioquímica, Bioquímica Clínica 1, Buenos Aires, Argentina; 2 YCARE (Youth/Child and cArdiovascular Risk and Evironmental) Research Group, Faculdade de Medicina, Universidade de Sao Paulo, São Paulo, SP Brazil; 3 Universidad de Antioquia. Escuela de Nutrición y Dietética, Medellin, Colombia; 4 DOMEN (MetabOlic Diseases, Exercise and Nutrition) Research Group, Center for Health Sciences, Federal University of Piaui, Teresina, Brazil; 5 University of Zaragoza, Faculty of Health Sciences – GENUD (Growth, Exercise, Nutrition and Development) Research Group, Instituto Agroalimentário de Aragón (IA2), Zaragoza, Spain; 6 Universidad Nacional Mayor de San Marcos, Faculty of Medicine, Department of Pediatrics, Universidad Peruana Cayetano Heredia, Research Associate Instituto Nacional de Salud del Niño, Lima, Peru; 7 Department of Epidemiology, School of Public Health, University of Sao Paulo, Sao Paulo, SP Brazil; 8 Universidad de Buenos Aires, CONICET, Facultad de Farmacia y Bioquímica, Buenos Aires, Argentina

**Keywords:** Biomarkers, Health care, Risk factors

## Abstract

Obesity and overweight in children and adolescents is increasing rapidly worldwide; however, scarce data have been reported from South America countries. With the purpose of assessing hyperlipidemia, insulin resistance and chronic inflammation, the evaluation of blood biomarkers such as glucose, lipoproteins and chronic inflammation proteins is required. In the context of the SAYCARE study, in children and adolescents (3 to 18 years) from seven South American cities, our aim was to assess the impact of pre analytical conditions on different biomarkers evaluated in 474 fresh serum samples, in different country centers. We also evaluated the stability according to time and frozen storage within this study across the concordance of the results obtained from the 49 blood samples measured in three different centers. Significant correlations as well as concordance were observed in TG, Total-C, HDL-C and glucose between Buenos Aires and São Paulo. The samples evaluated in Teresina and São Paulo presented similar results, with exception of total cholesterol. We observed acceptable concordance between Buenos Aires vs São Paulo and Teresina vs São Paulo, suggesting that samples could be processed in each of these centers. This concordance is a consequence of the strict pre analytical conditions previously established in the SAYCARE study.

## Introduction

With the purpose of assessing hyperlipidemia as well as insulin resistance and chronic inflammation, the evaluation of blood biomarkers such as glucose, lipids and lipoproteins as well as chronic inflammation proteins is required. The concentration of these parameters in blood samples depends on several factors, including those inherent to the individual, such as age and gender, and others referred to as pre analytical conditions. In clinical epidemiological multicenter studies, quality assurance in the pre analytical stage is essential for the precise interpretation of the results from the Clinical Biochemistry Laboratory^1^. Pre analytical conditions in these studies can be influenced by environmental factors, and those related to collection, handling, transportation, preparation and storage of diagnostic specimens^2^. In parallel, the methods used to obtain blood samples must be reliable, validated in the study population and comparable between countries. The requirements of the pre analytical and analytical stage are standardized in the guides of the World Health Organization (WHO)^3^ and the Clinical Laboratory Standards Institute (CLSI)^4^, and its recommendations must be achieved in order to reduce variability and errors.

To achieve our aims from SAYCARE, quality control is of great importance for obtaining reliable data and thus good results. For a multicenter study it is essential to ensure that the data collection is performed in a standardized way, thus achieving a good representation of reality and allowing comparisons of the data from the different cities involved in the study^5^.

Metabolic panel testing is the most routinely performed laboratory tests giving valuable information not only in enabling the diagnosis and directing further testing but also in monitoring patients^6^. In this regard, sample storage and transport are essential to ensure the quality of the clinical results^7^.

Recently, an observational multicenter study has been developed in seven cities from six countries of South America (Buenos Aires (Argentina), Lima (Peru), Medellin (Colombia), Montevideo (Uruguay), Santiago (Chile), São Paulo, and Teresina (Brazil)) in children and adolescents to assess lifestyle, cardiovascular health, and nutritional status, with standardized and jointly developed methods between countries. The South American Youth/Child Cardiovascular and Environmental (SAYCARE) study developed methods to collect reliable, comparable, and validated data about cardiovascular health biomarkers, lifestyles, and environmental, social, and familial factors^8–11^. In this context, the aim of our study was to assess the compliment of previously standardized pre analytical conditions for the obtainment of fresh samples of serum, in different country centers, across the SAYCARE Study. We also evaluated the stability of different biomarkers, according to transport and frozen storage within this pilot study, across the concordance of the results obtained from the same sample measured in different centers.

## Materials and Methods

### Study design

A detailed description of the SAYCARE sampling and recruitment methodology, data collection and quality control activities has been described^12^. Briefly, this is an observational cross-sectional school-based pilot study. Participants were in pre-school, primary school, and up to the third year of high school (3 to 18 years), enrolled in both public and private schools of their respective cities.

### Ethics

Procedures followed were in accordance with the ethical standards specified by the Helsinki Declaration of 1975, revised in 1983. The study protocol was presented to and accepted by the institutional Research Ethics Committee of the countries involved: Ethics Committee of the Faculty of Pharmacy and Biochemistry, University of Buenos Aires (Buenos Aires-Argentina), Ethics Committee on Human Research of the School of Medicine, University of São Paulo (São Paulo and Teresina, Brazil), Ethics Committee of the National Institute of Children Health (INSN) (Lima-Peru) and the Ethics Committee for Human Research of the University Research department (Medellin-Colombia). Parents of each participant gave their informed consent.

### Exclusion and inclusion criteria

The study’s exclusion criteria were pregnancy; inability to sign the informed consent of the parents, guardians and/or student. Subjects who, for other reasons not considered, should be eliminated after the study’ beginning, were also excluded. The study included all subjects between 3 and 18 years whose parents/guardians signed the informed consent form. Moreover, a signed assent form was obtained from all children/adolescents to indicate their approval to participate in the study.

### Sample collection

**Figure 1:**
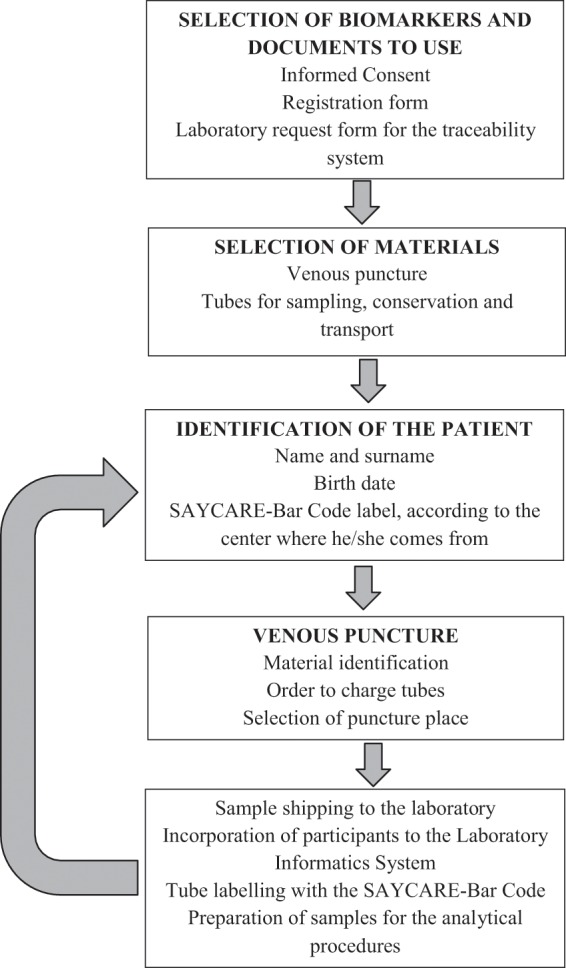
Procedure Sequence designed for the sampling, storage and shipping of blood samples among the different centers.

In order to ensure traceability and sampling conditions, previous meetings among the coordinators of each center were performed. A procedure sequence of the sampling and shipping process was design (Fig. 1). According to this, participants attended with 12 h fasting at school and samples were obtained between 8:30 and 9:00 am. Before sampling, each participant was identified with all the necessary data for incorporation into the laboratory information system (LIS), ie: full names, birth date and document number. The signing of informed consent authorized by their parents or a responsible adult was verified. The participants were in a seated position for the puncture; after selection of the puncture site, it was cleaned with 70% alcohol and the tourniquet was placed for less than three minutes, in order to avoid venous stasis and according to CLSI guideline^4^. Venous blood samples were collected by experienced phlebotomists; samples were taken through the same venipuncture site with butterfly system, in Vacutainer tubes system (Becton Dickinson, UK) with ethylenediaminetripotassium (EDTA K3, lilac cap) for hematologic determinations, and tubes with gel separator without additives for clinical chemistry determinations in serum (gold cap). Tubes were identified with a unique bar code in order to guarantee the traceability between the patient and the samples. The tube with gel separator without additives was first loaded, and then the tube with anticoagulant; both were fully loaded until exhausting the vacuum.

### Storage and transportation conditions

After sampling, tubes were placed in racks in vertical upright position in transportation boxes and stored in the dark, at 4 °C, and boxes were transported to the laboratory. Once in the laboratory samples were immediately deposited in the management samples area, registered in the LIS and sent to the analytical area. Samples were centrifuged for 15 min at 2200 g and 20 °C, within 30–90 min after sample collection. Hematologic and clinical chemistry determinations were performed immediately. Serum was divided in three aliquots, and stored in cryotubes. Two of them were analyzed in the country of origin, and the third tube was sent by a specialized transportation company, at −70 °C, to São Paulo for review and evaluation of reliability.

Twenty-seven serum samples from Buenos Aires and twenty two from Teresina, measured in the corresponding local laboratory, were also used to test the stability of different biomarkers during sample transport at the reference laboratory in São Paulo. To ensure traceability and optimal transport conditions from Buenos Aires and from Teresina to São Paulo, international guidelines such as Good Clinical Practices, Good Laboratory Practices, regulations of International Conference of Harmonization and the International Air Transport Association (IATA)^13^ were performed. In the reference laboratory, samples were measured in the same run.

### Biochemical analysis

Total cholesterol (Total-C), TG, HDL-C, fasting glucose and iron were measured using commercial enzymatic kits in autoanalyzer platforms of clinical chemistry. The coefficient of variation (CV) intra-assay <1.9%, CV inter-assay <2.4%, were in the average CV values of these parameters. LDL-C was calculated according to Friedwald^14^. Serum hs-CRP was determined by immunoturbidimetry assay in the same platform, CV intra-assay <1.9% and inter-assay <2.5% in Buenos Aires and Lima and by a high-sensitivity immunoturbidimetric assay using nephelometry, intra-assay CV <1.9% and inter-assay <2.5% in Teresina and São Paulo. VLDL-C was calculated as Total-C minus HDL-C and LDL-C. The more relevant indexes in clinical evaluation of lipoprotein profile were calculated: Total-C/HDL-C and TG/HDL-C (as a surrogate marker of insulin-resistance). Ferritin was evaluated by direct chemiluminometric technology. In all the centers, Riqas (Randox International Quality Assessment Scheme) external quality control was used.

### Statistical analysis

Data by center are presented as mean ± SD or median (range) according to normal or skewed distribution, respectively. Differences between groups were tested using the unpaired Student’s t test or the Mann Whitney U-test according to the data distribution. Pearson or Spearman analyses, for parametric or non-parametric variables, were used to determine correlations between parameters. To assess the degree of concordance in the Bland-Altman plot^15,16^, the bias was calculated for a 95% confidence interval. The SPSS 19.0 software package (Chicago, IL) and the Graph Pad Prism 5.01 software were used for statistical analysis. The criterion for statistical significance was set at 5%.

## Results

From a total of 1323 children and adolescents recruited in the SAYCARE study, 474 blood samples were collected with 12 h fasting, from 5 centers (108 in Buenos Aires-Argentina, 75 in São Paulo and 53 in Teresina-Brazil, 211 Medellin-Colombia, and 27 in Lima-Peru). Age and sex distribution of children and adolescents who were included in the study are depicted in Table 1.

**Table 1:** Distribution of the study population by sex.

Cities	Children		Adolescents	
	Male	Female	Male	Female
Buenos Aires	n = 31; 7 (6–10)	n = 30; 7 (3–10)	n = 20; 13 (11–16)	n = 27; 12 (11–16)
Lima	n = 4; 4 (3–5)	n = 6; 8(4–10)	n = 8; 14 (12–16)	n = 9; 15 (12–16)
Medellin	n = 42; 5 (3–10)	n = 98; 5(3–10)	n = 25; 17 (11–18)	n = 46; 16 (11–18)
São Paulo	n = 21; 5 (3–10)	n = 20; 5 (3–10)	n = 18; 13 (12–17)	n = 16; 14 (12–17)
Teresina	n = 13; 6(4–10)	n = 14; 7 (3–10)	n = 5; 15 (11–18)	n = 21; 12 (11–18)

Median age in years (range).

In each center samples were obtained under the same conditions, according to the designed procedure sequence. In Supplementary Files, glucose, lipoprotein profile, ferritin, iron and hs-CRP data can be observed. Similar results in serum concentrations of glucose and lipoprotein profile, in children and adolescents, were obtained among the different centers (Supplementary File 1). In a subgroup of children and adolescents from Buenos Aires, Lima, São Paulo and Teresina, ferritin, iron and hs-CRP were also evaluated (Supplementary File 2). Only minor differences between female and male from each center were observed in some parameters.

**Figure 2:**
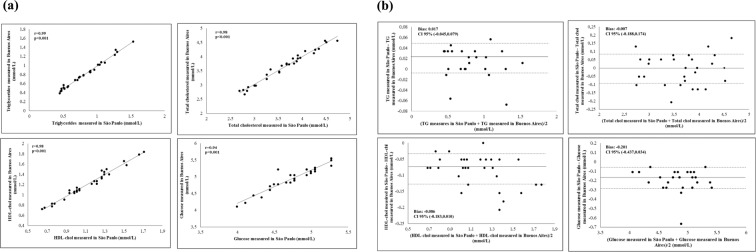
(**a**) Correlations of Triglycerides, total cholesterol, HDL-cholesterol and Glucose between Buenos Aires and São Paulo. (**b**) Concordance of Triglycerides, total cholesterol, HDL-cholesterol and Glucose between Buenos Aires and São Paulo.

**Figure 3:**
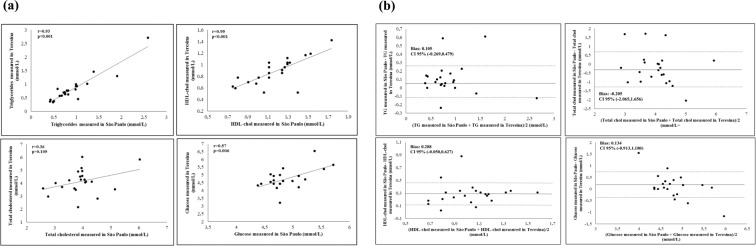
(**a)** Correlations of Triglycerides, total cholesterol, HDL-cholesterol and Glucose between Teresina and São Paulo. (**b**) Concordance of Triglycerides, total cholesterol, HDL-cholesterol and Glucose between Teresina and São Paulo.

The results of the stability tests of glucose, Total-C, TG and HDL-C performed in Buenos Aires and São Paulo, as well those performed in Teresina vs São Paulo are shown in Tables 2 and 3, and Figs. 2a,b and 3a,b.

**Table 2:** Stability tests of TG, Total-C, HDL-C and glucose performed in Buenos Aires and São Paulo.

	Mean level measured in Buenos Aires	Mean level measured in Sao Paulo	Mean difference	95% Confidence Interval
Triglycerides (mmol/L)	0.809	0.826	0.017	−0.045,0.079
Total-C (mmol/L)	3.627	3.620	−0.007	−0.188,0.174
HDL-C (mmol/L)	1.209	1.122	−0.086	−0.183,0.010
Glucose (mmol/L)	4.946	4.744	−0.201	−0.437,0.034

**Table 3:** Stability tests of TG, Total-C, HDL-C and glucose performed in Teresina and São Paulo.

	Mean level measured in Teresina	Mean level measured in Sao Paulo	Mean difference	95% Confidence Interval
Triglycerides (mmol/L)	0.830	0.935	0.105	−0.269,0.479
Total-C (mmol/L)	4.132	3.927	−0.205	−2.065,1.656
HDL-C (mmol/L)	0.886	1.175	0.288	−0.050,0.627
Glucose (mmol/L)	4.760	4.894	0.134	−0.913,1.180

When comparing the same sample in Buenos Aires and São Paulo, significant correlations were observed in TG, Total-C, HDL-C and glucose (Fig. 2a). An excellent concordance between these centers was observed. The Bland-Altman figure for these concordances can be seen in Fig. 2b. Regarding the correlations of parameters evaluated in the local laboratory of Teresina vs in São Paulo laboratory,TG, HDL-C and glucose directly correlated between both centers, while no correlation was found in Total-C between centers (Fig. 3a). Moderate concordances between these centers were obtained as seen in Fig. 3b.

According to these results, the storage and shipment of samples according to the international guidelines applied have slightly influence on the stability of the evaluated parameters.

## Discussion

In this study we assessed several analytical biomarkers, evaluated in blood samples from male and female children and adolescents from five South American countries, after applying a standardized protocol previously designed in accordance to the international guidelines for blood sample collection. We also show the concordance among different biomarkers, evaluated in the same sample in two different centers in comparison to the headquarter center.

In clinical epidemiological multicenter studies, quality assurance in the pre analytical stage is essential for the precise interpretation of the results from the Clinical Biochemistry Laboratory. The quality assurance and harmonization of all the procedures in this stage begin with managing the correct selection of biomarkers that will ensure the identification of risk factors or its association with metabolic disturbances, this is considered the “pre analytical stage”^2,17^. This stage, which also includes the drafting of the informed consent and the standardization of patient preparation, is patient-centered and is very important in order to reduce bias between different laboratories. With the purpose of reducing pre-analytical errors, investigators from each participating center were involved in the planning and development of the protocol, which included the study design. The identification and preparation of the patient and the sample collection, transport, handling and storage for the selected studies were previously coordinated according to international recommendations^17,18^. Prior to blood sampling, conditions pertinent to sample collection, such as fasting status, use of medications or physical activity, body posture during the sampling and type of sampling materials were standardized among the participant centers^19^.

Moreover, the quality of sample collected may be affected by conditions determined by different countries´ populations features, like ambient temperature, distance between the place of the specimen collection and the local laboratory, typical mealtimes or sleeping characteristics. In reference to the sample stability after collection, such as temperature, time until serum separation or processing and storage, documentation and shipping conditions were also to be specified and standardized^13^.

Some limitations must be considered; we analyzed concordance and correlations between samples from three centers, because only Buenos Aires and Teresina laboratories were able to send the samples to São Paulo. However, our sample size was sufficient to test the main objective of this paper; in a post-hoc sample size estimation analysis we proved that our sample was reliable to assess the effect of pre analytical conditions on the results after transportation. Moreover, the sample selection included private and public schools.

Regarding the standardization of these factors, an excellent concordance and correlation was obtained in all the evaluated parameters between Buenos Aires and São Paulo. Concerning the comparison between parameters measured in Teresina and São Paulo, concordance and correlations were slightly lower than those observed between Buenos Aires and São Paulo. The lack of correlation in Total-C between Teresina and São Paulo could be due to the selection of different reference standards in both centers, which causes a variation in the standardization of the assay. To improve the comparison between centers, the harmonization in the use of reference materials between all laboratories is recommended. However, given the excellent concordance observed between Buenos Aires and Teresina vs São Paulo, samples could also be processed in each of these centers. This concordance is a consequence of the strict pre analytical conditions previously established in the SAYCARE study. Our results suggest that money and time can be reduced in the blood samples analysis, and the results can be quickly informed to the patients because waiting for headquarter center to proceed the blood analysis is not necessary.

Regarding differences in some metabolic parameters among centers, most of them could be consequence of different food style (or eating habits), such as the higher Total-C and HDL-C observed in male from Buenos Aires in comparison to those from Lima and the variability in ferritin levels between centers^20,21^. Besides, other parameters could be affected by the different methodologies applied in each center, as in the case of hs-CRP, which, even though it did not surpass the reference value, it was clearly higher in Buenos Aires, in comparison to the other centers. Either way, it must be highlighted that in all the centers standardized methods with low variation coefficients were used.

Our study presents some limitations, such as the high rate of refusal to participate in the blood sample collection. Other limitation was the low number of samples that were contrasted with the reference laboratory in São Paulo. However, our results from the SAYCARE blood sample collection provide valuable results regarding future multicenter studies.

## Supplementary information

Dataset 1.
